# Isolation of *Limosilactobacillus mucosae* G01 with inhibitory effects on porcine epidemic diarrhea virus *in vitro* from Bama pig gastroenteritis

**DOI:** 10.3389/fmicb.2024.1360098

**Published:** 2024-08-07

**Authors:** Bin Zhang, Haiyan Shen, Hongchao Gou, Nile Wuri, Chunhong Zhang, Zhicheng Liu, Haiyan He, Jingjing Nie, Yunzhi Qu, Letu Geri, Jianfeng Zhang

**Affiliations:** ^1^Key Laboratory of Livestock Disease Prevention and Treatment of Guangdong Province, Institute of Animal Health, Guangdong Academy of Agricultural Sciences, Guangzhou, China; ^2^College of Veterinary Medicine, Inner Mongolia Agricultural University, Hohhot, China

**Keywords:** PEDV, antiviral activity, *L. mucosae* G01, IFN, Bama pig

## Abstract

Porcine epidemic diarrhea virus (PEDV) is responsible for causing fatal watery diarrhea in piglets, resulting in significant economic losses within the pig farming industry. Although vaccination is currently employed as a preventive measure, certain vaccines do not provide complete protection against PEDV field strains. Probiotics present a promising alternative due to their ability to regulate intestinal flora, enhance host immunity, and improve resistance against pathogenic microorganisms. We isolated six lactic acid bacteria (LAB) from the fecal microorganisms of Bama pigs, compared to *Limosilactobacillus mucosae* DSM13345 of the same genus in which *Limosilactobacillus mucosae* G01 (*L. mucosae* G01) proved to have a potent anti-PEDV effect. In a comprehensive manner, *L. mucosae* G01 significantly augmented the phosphorylation of IRF3 in IPEC-J2 cells, resulting in the induction of interferons (IFN α, IFN β, IFN λ1, and IFN λ3) and subsequent upregulation of interferon-stimulated genes (ISGs) (*MX1*, *MX2*, *OAS1*, and *ZAP*) in a dose-dependent fashion, consequently leading to the mitigation of PEDV replication. These findings underscore the promising prospects of *L. mucosae* G01 as a naturally derived substitute for combating PEDV and other enteric coronavirus infections.

## 1 Introduction

Piglet diarrhea has long been a significant challenge within the breeding industry, with the porcine epidemic diarrhea virus (PEDV) identified as the primary causative agent among various contributing factors. In particular, the infection of suckling piglets with PEDV often leads to severe diarrhea accompanied by a 100% mortality rate, resulting in significant economic losses (Jung et al., 2015; Kenney et al., 2021; Turlewicz-Podbielska and Pomorska-Mól, 2021). Although vaccination is commonly employed as a preventive measure, the effectiveness of certain vaccines may be compromised by genetic variations between the PEDV vaccine and the circulating strain in the field (Li et al., 2018; Luo et al., 2022). So, there is a need for developing complementary strategies that can prevent the spread of PEDV.

Probiotics have emerged as a promising alternative for restoring the ecological balance of the intestine and enhancing the intestinal microenvironment of the host (Kim et al., 2019). The beneficial effects of *Lactobacillus* on the gastrointestinal tract and overall health of the host are attributed to their ability to improve nutrient absorption, regulate harmful bacteria, and boost immune function (De Filippis et al., 2020; Di Giacomo et al., 2022). Additionally, the antiviral and antimicrobial properties of *Lactobacillus* further contribute to their potential as an alternative to conventional biological products (Arena et al., 2018). *Lactobacillus* has been found to disrupt viral adsorption and binding to epithelial surfaces, thereby inhibiting viral proliferation and stimulating immune responses against viruses (Wang et al., 2016; La Fata et al., 2018; Paone and Cani, 2020; Chen Y.-M. et al., 2022). Moreover, *Lactobacillus* has emerged as a leading microorganism in the colonization of the porcine intestinal tract, exerting a significant influence on the complex developmental trajectory of the intestinal immune system (Cebra, 1999). The strategic administration of *Lactobacillus* during episodes of gastrointestinal disturbance is crucial for maintaining the fragile balance of intestinal microflora (Tibary et al., 2006). This intervention provides a powerful method to protect the developing intestinal microbiota of nursing piglets during their critical early stages of growth and development (Siggers et al., 2008).

In comparison to other pig breeds in China, Bama sows exhibit enhanced adaptability and resistance to diseases, potentially due to the higher proportion of microbial species and greater abundance of gene copies of total bacteria in their gut microbiota (Yang et al., 2014). Previous studies have indicated the crucial involvement of intestinal microorganisms in the metabolic processes of Bama pigs, particularly during the pre-weaning stage (Yang et al., 2014; Ma et al., 2020; Wang K. et al., 2021). Chen C. et al. (2022) conducted an investigation into the intricate diversity of gut microbiota in Bama pigs, focusing on the effects of customized dietary interventions. The researchers discovered significant disparities in the composition of the gut microbiota in Bama pigs. By conducting thorough sequencing analysis of both gut flora and fecal microorganisms, they revealed a noteworthy abundance of probiotic species within the gastrointestinal ecosystem of these pigs. These findings serve as a relevant benchmark for studying probiotic populations present in the gut microbiota of Bama pigs (He et al., 2016; Chen C. et al., 2022). Consequently, these findings imply that the gut microbiota of Bama pigs might play a crucial role in their overall health and resistance to diseases.

In this study, our objective was to isolate *Lactobacillus* strains from Bama pig fecal and evaluate their capacity to inhibit PEDV replication *in vitro*. Through subsequent screening, we discovered a specific *Limosilactobacillus mucosae* G01 (*L. mucosae* G01) that exhibits antiviral properties. This *L. mucosae* G01 compound has the ability to enhance the phosphorylation of the crucial IRF3 protein in IPEC-J2 cells, leading to an upregulation of the transcription of type I and type III interferons, as well as interferon-stimulated genes (ISGs) such as *MX1*, *MX2*, *OAS1*, and *ZAP*. Consequently, this empowers IPEC-J2 cells to effectively combat PEDV infections.

## 2 Materials and methods

### 2.1 Cells and virus

The porcine intestinal epithelial cell line (IPEC-J2) were obtained from Dr. Li Wang at the Institute of Animal Science, Guangdong Academy of Agricultural Sciences, China. The cells were cultured in Roswell Park Memorial Institute (RPMI) Medium 1640 supplemented with 10% fetal bovine serum (FBS). Vero cells were stored in our laboratory at the Institute of Animal Health, Guangdong Academy of Agricultural Sciences, Guangzhou, China, were used for propagation of the GD/HZ/2016 (GenBank Accession: OP191700.1) strain. The vero cells were cultured in DMEM containing 15 μg/ml trypsin. The *L. mucosae* G01 strain uploaded with NCBI sequence number OR783178.

### 2.2 Isolation of *Lactobacillus* strains from fecal samples

The *Lactobacillus* strains utilized in this research were obtained from recently collected fecal samples of Guangxi Bama scented pigs. These samples were isolated from five sows in the early gestation stage and were collected over a span of three consecutive days. A total of 15 pig fecal samples were collected using sterile swabs. Subsequently, the samples were transferred into 1.5 mL centrifuge tubes containing PBS and promptly placed in ice boxes for transportation to the laboratory.

The procedures for isolating *Lactobacillus* strains from swine fecal samples were conducted following the methodology outlined by Halimi and Mirsalehian (2016). The collected fecal samples were introduced into de Man, Rogosa, and Sharpe (MRS) broth and incubated at a temperature of 37°C for a duration of 15 h. Subsequently, the samples were diluted and streaked onto MRS agar plates in triplicate. To amplify the 16S rRNA genes, the universal primers (27 F and 1492 R) were employed: 27F (5′-AGAGTTTGATCMTGGCTCAG-3′) and 1492R (5′- GGTTACCTTGTTACGACTT-3′). The PCR products obtained were ligated into the pMD19T vector and subsequently subjected to sequencing analysis using the automated sequencing service offered by Sangon Bioengineering (Guangzhou) Co., Ltd. The 16S rRNA sequences obtained were subjected to comparison using the MUSCLE algorithm in Mega 11 v11.0.13 (Callaway and Singh-Cundy, 2019). Subsequently, a phylogenetic tree was constructed employing the maximum Neighbor Joining (NJ) algorithm to elucidate the evolutionary relationships among the isolated strains. The resulting files were subsequently uploaded to iTOL^1^ (Shi et al., 2018) for the purpose of visualizing the evolutionary tree, which included species information for each strain.

### 2.3 Cell viability test of *Lactobacillus* strains

The cell viability assay of *Lactobacillus* strains on IPEC-J2 cells was conducted following modifications to the method described by EL-Adawi (El-Adawi, 2012). IPEC-J2 cell suspensions were prepared at a concentration of 5×10^4^ cells/ml and inoculated into 96-well plates, followed by incubation at 37°C for 12 h. Simultaneously, *Lactobacillus* strains were incubated overnight, washed three times with sterile PBS buffer, and subsequently centrifuged at 6,000 × *g* for 10 min. Subsequently, 10, 1, and 0.1 MOI of the *Lactobacillus* strains were added to each sample, which were then incubated for 24 h. The CCK-8 reagent was added and incubated at 37°C for 1 h. The absorbance at a wavelength of 450 nm was quantified utilizing the Multiskan Sky instrument manufactured by Thermo Scientific. The result of the calculation is: Cell viability (%) = (ODt / ODc) × 100%, where ODt and ODc are the absorbance of treated and control cells, respectively (Kurpe et al., 2020).

### 2.4 *In vitro* evaluation of PEDV with *Lactobacillus* strains

The experimental methodology employed to evaluate the therapeutic efficacy of *Lactobacillus* strains on IPEC-J2 cells infected with the PEDV strain GD/HZ/2016 was derived from the work of Chang-Liao et al. (2020). To assess the anti-PEDV effect, the IPEC-J2 cells were subjected to both prestimulation and poststimulation using *Lactobacillus* strains. For the prestimulation phase, the IPEC-J2 cells were cultured in 12-well plates and exposed to either 1 or 0.1 MOI of *Lactobacillus* strains. Following a 2 h incubation period, the cells were rinsed thrice with sterile PBS and subsequently infected with 0.1 MOI of PEDV. Post-stimulation, the IPEC-J2 cells were exposed to 0.1 MOI of PEDV, followed by three washes with sterile PBS. Subsequently, the cells were treated with either 1 or 0.1 MOI of *Lactobacillus* strains for a duration of 2 h. After another wash with sterile PBS, the cultures were incubated at 37°C in a 5% CO_2_ environment and harvested 24 h post-infection with PEDV. Cell samples were collected for qRT-PCR analyses to evaluate the impact of *Lactobacillus* strains on virus replication.

### 2.5 RNA extraction and reverse transcription quantitative polymerase chain reaction (RT-qPCR)

The TaKaRa MiniBEST Universal RNA Extraction Kit, manufactured in China by TaKaRa, was utilized to extract total RNA from IPEC-J2 cells subjected to various experimental conditions. For qRT-PCR analysis, the HiScript^®^ II one Step qRT-PCR SYBR Green Kit, produced by Vazyme in China, was employed. The primer sequences employed for gene detection can be found in Table 1.

**TABLE 1:** Amplification of RT-qPCR using specific primers.

Gene	Forward primer (5′-3′)	Reverse primer (5′-3′)
β-actin	CCGCACCACTGG CATTGTC	CTCCTTGATGTCC CGCACG
IFN-α	GCCTCCTGCACCAG TTCTACA	TGCATGACACAG GCTTCCA
IFN-β	TAGGCGACACTGTTC GTGTTG	CCAAGCAAGTTGTA GCTCATGG
IFN-λ1	GCCAGTTCCAAT CTCTGT	CCTTAGGACCTTCA GAGTCA
IFN-λ3	GTGCCTGTCCCT GAAGC	CTGCTCCTGGGG AAGATG
MX1	GCAGCCAGTACGAGG AGAAG	CTCCTGACAGTGC CTCCAAC
ISG15	GGGCAACGAGTTC CAGGT	CACCACCAGCAGG ACCGT
OAS1	GGTTGTCTTCCTCAG TCCTC	AGCCTGGACCTCA AACTTCA
PEDV N	GCAAAGACTGAACC CACTAAT	GCCTCTGTTGT TACTTGGAG
MX2	TTCACTCGCATCCGCA CTTCAG	AGCTCCTCTGTCG CACTCTGG
ZAP	GCTCAGTGCGAACACC TGGATG	TGACAGATGAAGG CGTGGAGAGG

### 2.6 Western blot

The experimental procedure was performed according to the methodology outlined by Shen et al. (2016). In brief, IPEC-J2 cells were lysed using 150 μL of lysis buffer obtained from Thermo Fisher Scientific, China. The resulting proteins were then separated and transferred onto a polyvinylidene fluoride membrane. Subsequently, the membrane was incubated for 1 h with 5% non-fat milk, followed by a 2 h incubation with primary antibodies against PEDV N-protein (Medgene Labs, USA), glyceraldehyde-3-phosphate dehydrogenase (GAPDH) (ABclonal, China), synthetic phosphorylated peptide around interferon regulatory Factor 3 (pIRF3) (ABclonal, China), and interferon regulatory Factor 3 (IRF3) (ABclonal, China), followed by three washes with PBST. The PVDF membranes were subsequently subjected to incubation with horseradish peroxidase-conjugated goat anti-mouse and rabbit anti-lgG(H + L) secondary antibodies (Bioworld, China). The visualization of immunity protein bands was achieved by employing an ECL Kit (Millipore, China).

### 2.7 Immunofluorescence assay

Refer to Zhang W’s method (Zhang et al., 2023) as follows. The cells were fixed using a 4% paraformaldehyde solution (Beyotime, China) and permeabilized with a PBS solution containing 0.1% Triton X-100. Subsequently, the cells were blocked with a 1% bovine serum albumin solution (BBI Life Sciences, China) for 1 h. Following this, the cells were incubated overnight at 4°C with a mouse anti-PEDV N IgG antibody, and then with Alexa Fluor™ 594 goat anti-mouse IgG (Invitrogen, China) for 1 h at 37°C. After washing with PBS three times, the cells were incubated with 4,6-diamidino-2-phenylindole (DAPI, Beyotime) for 5 min. Finally, the cells were examined using a differential fluorescence microscope.

### 2.8 Virus titer detection

Refer to Zhang et al. (2023) method as follows. In order to ascertain the virus titers, Vero cells were cultivated in 96-well plates until reaching a confluence of 70–80%. Subsequently, the cells were infected with 10-fold serial dilutions of PEDV, with a total of four replicates. Following an incubation period of 72 h, the cells were evaluated using the immunofluorescence assay (IFA). The Reed-Muench method was employed to determine the virus titers, which were quantified as TCID_50_ per milliliter.

### 2.9 Data analysis

The data analysis was performed utilizing GraphPad Prism v8.0 software, employing an ANOVA to evaluate the statistical significance of differences in mean values. The mean values for each group were reported as the mean ± standard deviation (SD). Statistical analyses were conducted to ascertain the presence of significant differences between the treatment and control groups. One-way analysis of variance followed by the Tukey-Kramer post-test was used. A *p* < 0.05 was considered statistically significant.

## 3 Results

### 3.1 Molecular identification of isolated *Lactobacillus* strains from Bama pig fecal

**FIGURE 1:**
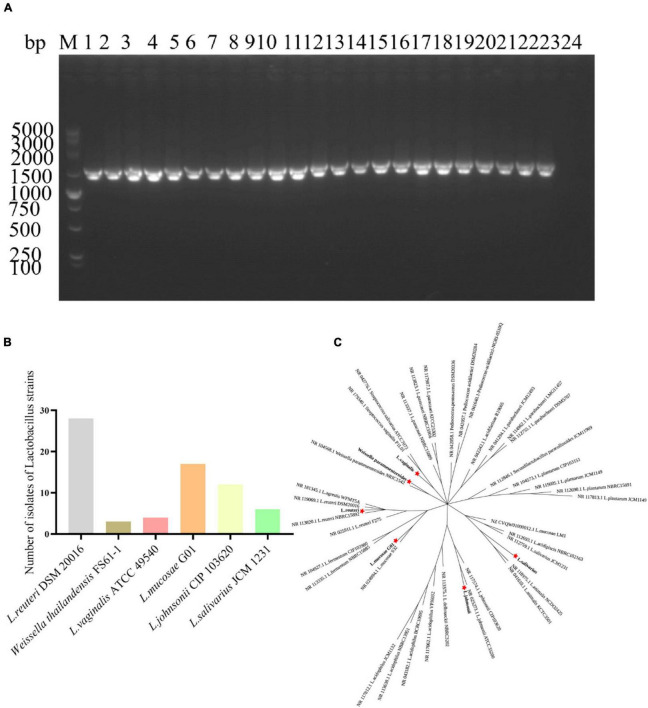
Isolation and characterization of *Lactobacillus* strains from fecal samples of Bama pigs. **(A)** Representative amplification of the PCR product using primers 27F/1492R. M denotes the DL5000 marker, and 1–23 correspond to the monoclonal *Lactobacillus* strains tested. The Line 24 was used as a negative control well. **(B)** Proportion of total isolates accounted for by *Lactobacillus* in fecal samples. Gray color is *L. reuteri* DSM20016 strain, brown color is *Weissella thailandensis* FS61-1 strain, light pink color is *L. vaginalis* ATCC49540 strain, orange color is *L. mucosae* G01, light yellow color is *L. johnsonii* CIP103620, and green color is *L. salivarius* JCM1231 strain. **(C)** An evolutionary tree was constructed employing the maximum likelihood method, utilizing 16S rRNA gene sequences of the isolated *Lactobacillus* strains. Mega 11 v11.0.13 was employed for construction, and the tree was enhanced using the iTOL online software. The red star pattern indicates the current isolate.

A total of 70 *Lactobacillus* strains were selected for polymerase chain reaction (PCR) using the 27F/1492R common primer specific for bacterial 16S rRNA. This PCR amplification resulted in a bright band at approximately 1500 base pairs, indicating successful amplification of the 16S rRNA product (Figure 1A). The PCR products were subsequently sequenced and compared to the National Center for Biotechnology Information (NCBI) database for further analysis of the isolated *Lactobacillus* species. The analysis revealed that 28 strains potentially belonged to *Limosilactobacillus reuteri* DSM 20016 (*L. reuteri* DSM20016), 3 strains to *Weissella thailandensis* FS61-1, and 4 strains to *Limosilactobacillus vaginalis* ATCC 49540 (*L. vaginalis* ATCC49540). Seventeen strains were identified as *Limosilactobacillus mucosae* G01 (*L. mucosae* G01), twelve as *Limosilactobacillus johnsonii* CIP103620 (*L. johnsonii* CIP103620), and 6 strains as *Ligilactobacillus salivarius* JCM1231 (*L. salivarius* JCM1231) (Figure 1B). The construction of a phylogenetic tree utilizing the 16S rRNA gene sequences revealed a range of *Lactobacillus* species that were obtainable from Bama pigs fecal (Figure 1C).

### 3.2 Cytotoxicity test of *Lactobacillus* strains

**FIGURE 2:**
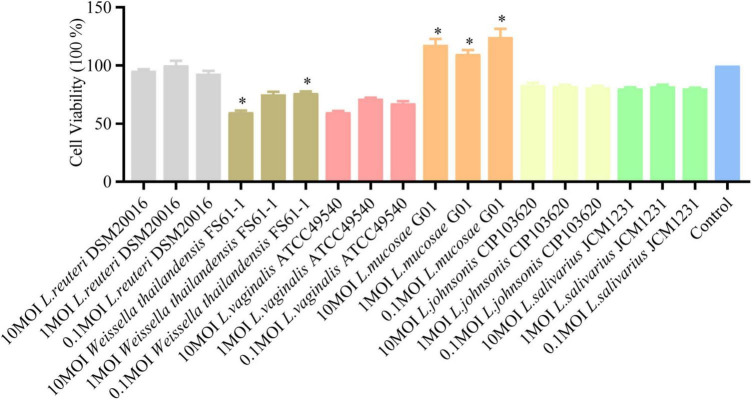
Effect of isolated lactic acid bacteria strains on IPEC-J2 cell viability. *Lactobacillus* strains, isolated from intestinal and fecal samples, were incubated with IPEC-J2 cells at concentrations of 10, 1, and 0.1 MOI for 24 h at 37°C, and cell viability was assessed using the CCK-8 (5 mg/ml, 10 μl/ well). OD_450_, a widely employed method for measuring cell viability, is presented. Gray color is *L. reuteri* DSM20016 strain, brown color is *Weissella thailandensis* FS61-1 strain, light pink color is *L. vaginalis* ATCC49540 strain, orange color is *L. mucosae* G01, light yellow color is *L. johnsonii* CIP103620, green color is *L. salivarius* JCM1231 strain and blue color is IPEC-J2 cells. All assays were performed in triplicates, with three replicates per experiment, and each bar is the mean ± SEM. The asterisk indicates significant differences compared to the control group (*p* < 0.05).

To assess the cytotoxic effects of various *Lactobacillus* strains on IPEC-J2 cells, a CCK-8 assay was conducted using three different bacterial concentrations (10, 1, and 0.1 MOI). As illustrated in Figure 2, the *L. mucosae* G01 strain was observed to promote the proliferation of IPEC-J2 cells, as evidenced by the optical density value (OD_450_) detected using the CCK-8 reagent. At a concentration of 0.1 MOI, *L. johnsonii* CIP103620, *L. salivarius* JCM1231, and the *L. mucosae* G01 strain exhibited no cytotoxicity toward IPEC-J2 cells. Conversely, *Weissella thailandensis* FS61-1 and *L. vaginalis* ATCC49540 demonstrated cytotoxic effects on IPEC-J2 cells.

### 3.3 Screening of antiviral *Lactobacillus* strains

**FIGURE 3:**
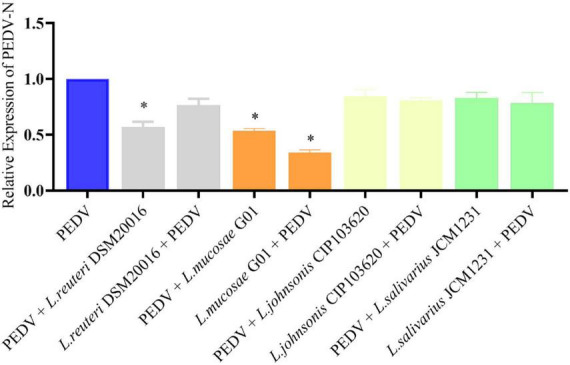
*Lactobacillus* strains screened for antiviral activity. IPEC-J2 cells were pre-stimulated or post-stimulated with *Lactobacillus* strains at 1 MOI for 2 h before or after PEDV infection. Following incubation at 37°C for 24 h, the copy number of the PEDV N gene was quantified using quantitative reverse transcription polymerase chain reaction (qRT-PCR). Gray color is *L. reuteri* DSM20016 strain, orange color is *L. mucosae* G01, light yellow color is *L. johnsonii* CIP103620, green color is *L. salivarius* JCM1231 strain and blue color is PEDV control group. All assays were performed in triplicates, with three replicates per experiment, and each bar is the mean ± SEM. The asterisk indicates significant differences compared to the control group (*p* < 0.05).

The inhibitory effects of *L. reuteri* DSM20016, *L. mucosae* G01, *L. johnsonii* CIP103620, and *L. salivarius* JCM1231 on PEDV infection in IPEC-J2 cells were assessed through prestimulation and poststimulation (Figure 3). Analysis using qRT-PCR revealed a significant reduction in PEDV N mRNA levels with *L. mucosae* G01 treatment. Furthermore, *L. reuteri* DSM20016 and *L. mucosae* G01 demonstrated inhibition of viral replication in the poststimulation mode, while the other strains did not exhibit a significant effect. Considering the antiviral properties observed, *L. mucosae* G01 was chosen for subsequent investigation.

### 3.4 Comparison of the anti-PEDV effect of *L. mucosae* G01 with that of commercialized *L. mucosae* DSM13345

**FIGURE 4:**
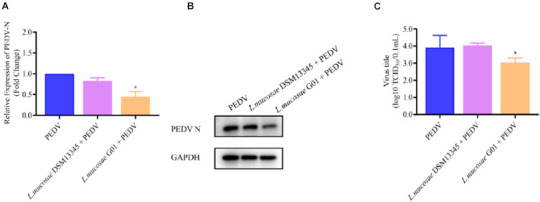
Depicts a comparison of the anti-PEDV effects of 1 MOI *L. mucosae* DSM13345 and *L. mucosae* G01 strains on IPEC-J2 cells. The method was utilized to evaluate the expression levels of the N protein in *L. mucosae* DSM13345 and *L. mucosae* G01 following 2 h interaction with IPEC-J2 cells, subsequent addition of PEDV-HZ, and after 24 h cell supernatants were harvested. Cell lysate containing PMSF was added for nucleic acid extraction and Western Blot assay. **(A)** RT-qPCR method was used to detect *L. mucosae* DSM13345 and *L. mucosae* G01 samples PEDV N mRNA expression level. **(B)** The quantification of N protein expression levels was performed through Western blot analysis. PEDV N is the viral content of the N nucleocapsid, and GAPDH is an internal reference for harvested cells. **(C)** TCID_50_ analysis cell supernatant titer. The purple color is *L. mucosae* DSM13345, orange color is *L. mucosae* G01, and blue color is PEDV control group. All assays were performed in triplicates, with three replicates per experiment, and each bar is the mean ± SEM. The asterisk indicates significant differences compared to the control group (*p* < 0.05).

In order to assess the anti-PEDV efficacy of various *L. mucosae* G01 strains, the *L. mucosae* DSM13345 strain (obtained from Minebea) was utilized as a control. IPEC-J2 cells infected with PEDV were subjected to pre-stimulation with *L. mucosae* at a 1 MOI concentration, and the expression levels of PEDV N mRNA were determined using qPCR. The *L. mucosae* G01 strain isolated from the intestine demonstrated a significant inhibitory effect on PEDV replication in comparison to the *L. mucosae* DSM13345 strain (Figure 4). The *L. mucosae* G01 significantly reduced (*p* < 0.05) PEDV N mRNA expression compared to *L. mucosae* DSM13345 (Figure 4A), and supernatant viral titers were shown to be consistent with mRNA levels as well (Figure 4C). The findings from the figure strongly suggest that *L. mucosae* G01 possesses the potential to counteract PEDV infection in IPEC-J2 cells.

**FIGURE 5:**
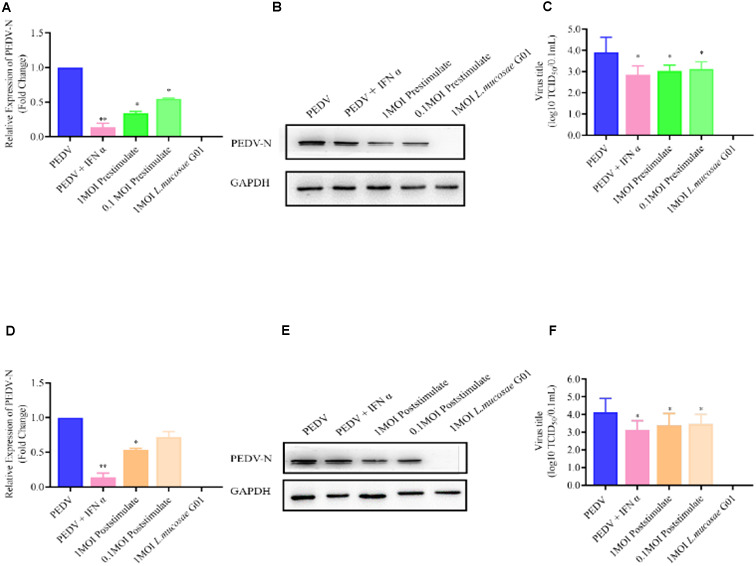
*L. mucosae* G01 inhibits PEDV replication with both prestimulate and post stimulate. Depicts the prestimulation and poststimulation effects of *L. mucosae* G01 strain were demonstrated in IPEC-J2 cells after 24 h with 1 and 0.1 MOI and expression level of PEDV N protein and treat vero cells with a 10-fold dilution of viral supernatant for 72 h. **(A)** Expression content of mRNA levels of PEDV N nucleocapsid protein in response to prestimulation at 1 and 0.1 MOI. **(B)** Expression levels of PEDV N protein in response to prestimulation with at 1 and 0.1 MOI. **(C)** TCID_50_ analysis cell supernatant titer were measured with prestimulation at 1 and 0.1 MOI. **(D)** Expression content of mRNA levels of PEDV N nucleocapsid protein in response to poststimulation at 1 and 0.1 MOI. **(E)** Expression levels of PEDV N protein in response to poststimulation with at 1 and 0.1 MOI. **(F)** TCID_50_ analysis cell supernatant titer were measured with prestimulation at 1 and 0.1 MOI. The experimental groups included PEDV-infected, virus infected after IFN treated cells, cells infected with *L. mucosae* G01 1 MOI and then treated with PEDV, cells infected with *L. mucosae* G01 0.1 MOI and then treated with PEDV, and cells infected with *L. mucosae* G01. Cells infected 0.1 MOI PEDV then treated with *L. mucosae* G01 at 1 and 0.1 MOI. Pink color is PEDV and IFN-α as positive control. Green is the prestimulation group: 1 MOI *L. mucosae* G01 interacted with IPEC-J2 cells followed by 0.1 MOI PEDV, light green is the prestimulation group: 0.1 MOI *L. mucosae* G01 interacted with IPEC-J2 cells followed by 0.1 MOI PEDV, and orange is the treatment group: 0.1 MOI PEDV interacted with IPEC-J2 cells before interacting with 1 MOI *L. mucosae* G01, light yellow is 0.1 MOI PEDV first interacting with IPEC-J2 cells before interacting with 0.1 MOI *L. mucosae* G01 and blue color is PEDV control group. All assays were performed in triplicates, with three replicates per experiment, and each bar is the mean ± SEM. Statistically significant differences between groups are indicated by *p < 0.05 or **p < 0.01.

To ascertain the potential correlation between the concentration of *L. mucosae* G01 and the potency of antiviral activity, our findings indicate that pre-stimulating IPEC-J2 cells with 0.1 and 1 MOI of *L. mucosae* G01 for a duration of 24 h resulted in a notable reduction in PEDV N mRNA levels (*p* < 0.05) (Figure 5A). Furthermore, a dose-dependent decline in PEDV N protein expression was observed (*p* < 0.05) (Figures 5B, E). These outcomes strongly imply that *L. mucosae* G01 effectively impedes PEDV production, with inhibition rates ranging from 21.90 to 68.10%. Furthermore, our study revealed a significant decrease in PEDV titers in a dose-dependent manner (*p* < 0.05), as evidenced by a 1.53-log reduction in progeny virus levels, indicating the inhibition of PEDV replication (Figures 5C, F). However, no statistically significant differences were observed in the expression levels of PEDV N mRNA in poststimulation IPEC-J2 cells following PEDV infection at 0.1 MOI *L. mucosae* G01 (Figure 5D). These findings suggest that *L. mucosae* G01 exhibits anti-PEDV activities, which hold potential for the development of effective interventions against this pathogen.

### 3.5 The *L. mucosae* G01 strain induces interferon and interferon-stimulated genes (ISGs) production in IPEC-J2 cells

**FIGURE 6:**
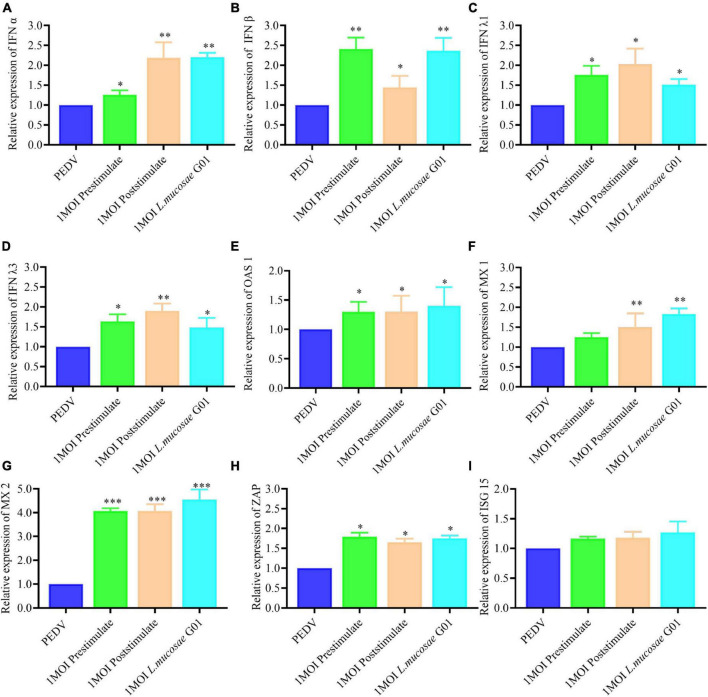
The *L. mucosae* G01 strain induces interferon and Interferon-stimulated genes (ISGs). The prestimulation and poststimulation effects of *L. mucosae* G01 strain were demonstrated in IPEC-J2 cells after 24 h with 1 MOI. Cells were harvested and cell lysate containing PMSF was added, followed by nucleic acid extraction. The mRNA levels of IFN α, IFN β, IFN λ1, IFN λ3 and effector protein were detected using qPCR. **(A)** Expression content of mRNA levels of IFN α in response to prestimulation or poststimulation at 1 MOI. **(B)** Expression content of mRNA levels of IFN β in response to prestimulation or poststimulation at 1 MOI. **(C)** Expression content of mRNA levels of IFN λ1 in response to prestimulation or poststimulation at 1 MOI. **(D)** Expression content of mRNA levels of IFN λ3 in response to prestimulation or poststimulation at 1 MOI. **(E)** Expression content of mRNA levels of *OAS1* in response to prestimulation or poststimulation at 1 MOI. **(F)** Expression content of mRNA levels of *MX1* in response to prestimulation or poststimulation at 1 MOI. **(G)** Expression content of mRNA levels of *MX2* in response to prestimulation or poststimulation at 1 MOI. **(H)** Expression content of mRNA levels of *ZAP* in response to prestimulation or poststimulation at 1 MOI. **(I)** Expression content of mRNA levels of *ISG15* in response to prestimulation or poststimulation at 1 MOI. The experimental groups included PEDV infected, cells infected with *L. mucosae* G01 1 MOI and then treated with PEDV, cells infected with *L. mucosae* G01 1 MOI and then treated with PEDV, and cells infected with *L. mucosae* G01. Green is the prestimulation group: 1 MOI *L. mucosae* G01 interacted with IPEC-J2 cells followed by 0.1 MOI PEDV, orange is the treatment group: 0.1 MOI PEDV interacted with IPEC-J2 cells before interacting with 1 MOI *L. mucosae* G01, azure is the *L. mucosae* G01 group, and blue color is PEDV control group. All assays were performed in triplicates, with three replicates per experiment, and each bar is the mean ± SEM. Statistically significant differences between groups are indicated by *p < 0.05, **p < 0.01 and ***p < 0.001.

In the present study, the investigation focused on assessing the levels of interferons (IFN α, IFN β, IFN λ1, and IFN λ3) in IPEC-J2 cells before and after stimulation with the *L. mucosae* G01 strain. The obtained results revealed a significant elevation in the expression of IFN types of interferons in IPEC-J2 cells inoculated with *L. mucosae* G01 compared to the control group. Furthermore, both pre- and post-stimulation with *L. mucosae* G01 were found to significantly enhance the expression of IFN α, IFN β, IFN λ1, and IFN λ3 (Figures 6A–D). These findings suggest that *L. mucosae* G01 has the potential to augment the antiviral response by inducing the expression of interferons in IPEC-J2 cells.

When comparing the IPEC-J2 cells after stimulation with *L. mucosae* G01 to the PEDV cells, it was observed that both prestimulation and poststimulation resulted in a significant increase in *MX2* expression (*p* < 0.001) (Figure 6G). Furthermore, the levels of *OAS1*, *MX1*, and *ZAP* expression in the prestimulated and post stimulated IPEC-J2 cells were found to be significantly different following *L. mucosae* G01 infection (*p* < 0.05) (Figures 6E, F, H). However, *L. mucosae* G01 did not cause changes in ISG15 (Figure 6I).

### 3.6 The *L. mucosae* G01 strain can stimulate IRF3 phosphorylation in IPEC-J2 cells

PEDV binds to the TLR3 receptor in IPEC-J2 cells, triggering an intracellular signaling cascade that results in the phosphorylation of interferon regulatory factor 3 (IRF3). Subsequently, the activated IRF3 translocates to the nucleus and stimulates the transcription of interferons (Stanifer et al., 2019).

**FIGURE 7:**
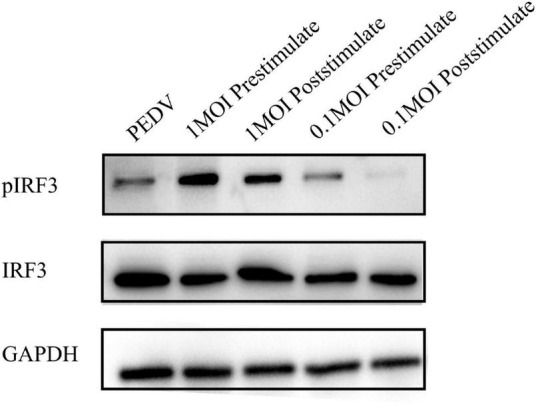
The *L. mucosae* G01 strain upregulates stimulate IRF3 phosphorylation in IPEC-J2 cells. The pIRF3 is phosphorylated IRF3, pIRF3/IRF3 ratio assesses whether the interferon pathway is activated, and GAPDH, an intracellular reference. The *L. mucosae* G01 strain at 1 and 0.1 MOI were demonstrated in IPEC-J2 cells after 30 min. IPEC-J2 cells were pre-treated with 1 and 0.1 MOI of *L. mucosae* G01 for 30 min, followed by the addition of 0.1 MOI PEDV, and harvested after 2 h. Western blot analysis was performed to quantify the levels of p-IRF3/IRF3 after prestimulation and poststimulation the cells with PEDV. The different treatment conditions included PEDVgroup, *L. mucosae* G01 1 MOI treated with PEDV, PEDV treated with *L. mucosae* G01 1 MOI, *L. mucosae* G01 0.1 MOI treated with PEDV, and 0.1 MOI *L. mucosae* G01 treated with PEDV.

To investigate the impact of *L. mucosae* G01 on IRF3 phosphorylation and IFN signaling, we conducted an assessment of the pIRF3/IRF3 ratio in IPEC-J2 cells subjected to *L. mucosae* G01 treatment. The outcomes demonstrate that exposure to 1 MOI and 0.1 MOI *L. mucosae* G01 strain resulted in a dose-dependent stimulation of IRF3 phosphorylation in IPEC-J2 cells (Figure 7). These findings imply that *L. mucosae* G01 possesses the ability to induce IRF3 phosphorylation in IPEC-J2 cells, potentially contributing to its anti-PEDV properties. The quantification of p-IRF3/IRF3 levels was accomplished through western blot analysis of lysed cellular proteins. The results of this study indicate that *L. mucosae* G01 potentially contributes to the activation of IFN signaling and the induction of IRF3 phosphorylation in IPEC-J2 cells.

## 4 Discussion

Since 2010, China has experienced a widespread outbreak of PEDV, which has had significant clinical implications for newborn piglets and resulted in substantial economic losses within the swine industry (Vlasova et al., 2014; Sujie et al., 2022). In recent years, there has been an increasing number of studies advocating for the use of probiotics to regulate the balance of intestinal flora and reduce piglet mortality (Inatomi et al., 2017; Wang et al., 2017; Hou et al., 2018). The Bama pig, a unique Chinese breed known for its strong disease resistance and diverse gut microbiota, has been subject to screening of *Lactobacillus* strains from piglet fecal samples, which has been reported to significantly improve the growth performance of weaned piglets (Wang W. et al., 2021). In this study, a total of six *Lactobacillus* strains were isolated from fecal samples obtained from Bama-scented pigs. The *Lactobacillus* species accounted for 61.95% of all isolates. The findings indicate that the relative abundance of *Lactobacillus* species in wild boar fecal was higher compared to that in domestic pigs, with *L. mucosae* G01 representing 54.54% of the total isolates (Zhong et al., 2022). The successful isolation and verification of *L. mucosae*’s widespread presence in Bama pigs provide a solid foundation for utilizing it as a probiotic food additive. Furthermore, probiotics play a crucial role in acting as a protective barrier against pathogenic microorganisms within the intestinal tract. Li et al. (2022) discovered that local specialty breeds of hogs exhibited a more diverse microflora population in the corresponding intestinal segments when compared to large white hogs. Notably, significant variations were observed in the abundance of *L. mucosae* G01 and Streptococcus equi. Furthermore, our isolated *L. mucosae* G01 demonstrated a remarkable 1.53-log reduction in PEDV activity, surpassing the effectiveness of commercially available *L. mucosae* DSM13345. The unique characteristics of this strain may be attributed to long-term dietary and environmental disparities among pigs. The study conducted by Huang et al. (2023) demonstrated that *L. reuteri*, obtained from the fecal of large white pigs, exhibited significant superiority in preventing, treating, and competing against PEDV, particularly the *L. reuteri* LRM strain. These findings align with our own assessment, conducted in a comparative manner.

Huang et al. (2023) conducted a study in which they employed *L. reuteri*, isolated from pig fecal, and observed a decrease in the mRNA expression of inflammatory cytokines. This methodology demonstrated efficacy in alleviating clinical symptoms and intestinal damage in piglets infected with PEDV. Additionally, Chang-Liao et al. (2020) performed an *in vitro* evaluation to assess the effectiveness of *Ln. mesenteroides*, derived from kefir grains. In contrast to the aforementioned approach, the *Ln. mesenteroides* strain exhibits direct interaction with PEDV *in vitro*, subsequently exerting an inhibitory effect on PEDV replication in Vero cells through neutralization of the viral form. Our dissimilarities stem from the bacteria’s capacity to occupy receptors on the cell surface in the simulated animal’s natural state, thereby impeding PEDV invasion into the organism, or the bacterial modulation of the cell itself to elicit a mechanistic response against PEDV subsequent to its invasion into the organism. It is noteworthy that the aforementioned favorable results were primarily observed in vero cells, yet there is a lack of lactobacilli evaluation regarding PEDV invasion in intestinal epithelial cells. The present study aimed to address this gap by conducting *in vitro* investigations, which revealed that *L. mucosae* G01 induced the activation and subsequent phosphorylation of the IRF3 protein. The subsequent activation resulted in an increase in the mRNA expression of IFN-I and IFN-III genes, alongside the induction of antiviral proteins including *MX2*, *OAS1*, and *ZAP*. These findings provide insights into the immunomodulatory function of *L. mucosae* G01 during PEDV infection.

During the initial phases of viral infections, interferons play a pivotal role in the host’s defense mechanism against pathogenic microorganisms. Both type I and type III interferons stimulate the expression of antiviral genes within cells, thereby enhancing their ability to withstand viral infections (Lazear et al., 2015; Stanifer et al., 2019). Despite their distinct receptors and secreted cell types, these interferons facilitate the natural immune response, activate immune cells, and provide protection against pathogenic infections (Mesev et al., 2019). Type I interferon is primarily secreted by infected and immune cells, whereas type III interferon is predominantly secreted by epithelial and specific immune cells, particularly on mucosal surfaces and within epithelial cells, thereby regulating viral infection (Li et al., 2017; Mesev et al., 2019). In the interferon signaling pathway, IRF3 plays a crucial role as it becomes phosphorylated and translocated to the nucleus upon PEDV infection, facilitating the transcription of both type I and type III interferons (Stanifer et al., 2019; Su et al., 2020). *Lactobacillus* strains possess the capacity to modulate the immune response within the intestines and combat pathogenic microorganisms by stimulating IRF3 and promoting the production of type I interferon (Gonzalez-Ochoa et al., 2017). Prior research has demonstrated that lactobacilli activate IRF3 and induce IFN-I expression via the Toll-like receptor pathway (Kanmani and Kim, 2019). Moreover, lactobacilli play a role in activating the innate immune system of intestinal epithelial cells to defend against viral intrusion. Our study provides evidence that *L. mucosae* G01 has the ability to induce the expression of IRF3 protein and enhance the secretion of both IFN-I and IFN-III, resulting in the upregulation of antiviral proteins *MX1*, *MX2*, *OAS1*, and *ZAP*. In the post-stimulation trial, the reduction in *L. mucosae* G01 virus can be attributed to the activation of the IFN signaling pathway, which facilitates the clearance of the virus by enhancing cellular antiviral responses. Several studies have provided support for our findings. In addition, the research conducted by Kanmani et al. has demonstrated that *L. delbrueckii* TUA4408L possesses the ability to impede rotavirus replication by activating IRF3 (Kanmani et al., 2018). Furthermore, administering probiotics to rats has been found to enhance the expression of interferon-induced *MX1* mRNA (Yu et al., 2010). In the forthcoming stage of our research program, we will commence a comprehensive investigation into the *in vivo* verification of the antiviral attributes of *L. mucosae* G01. This crucial research endeavor aims to elucidate the degree of safeguarding that probiotics can bestow upon the gastrointestinal wellbeing of piglets, thereby augmenting our comprehension of their potential as an invaluable mechanism in mitigating PEDV-associated difficulties.

In summary, our research demonstrates the notable capacity of *L. mucosae* G01 to hinder PEDV replication through dual mechanisms, involving direct viral inhibition and the activation of the IFN signaling pathway *in vitro*. These findings have substantial implications for the development of strategies targeting the prevention and control of PEDV infections.

## Data availability statement

The data presented in the study are deposited in the NCBI Nucleotide repository, accession number GenBank: OR783178.1.

## Ethics statement

The approval for animal experiments was obtained from the Experimental Animal Ethics Committee of Institute of Animal Health, Guangdong Academy of Agricultural Sciences with the SYXK(Yue)X2021-0165. The studies were conducted in accordance with the local legislation and institutional requirements. Written informed consent was obtained from the owners for the participation of their animals in this study.

## Author contributions

BZ: Writing – original draft. HS: Writing – review & editing. HG: Writing – review & editing. NW: Methodology, Writing – review & editing. CZ: Project administration, Writing – review & editing. ZL: Project administration, Writing – review & editing. HH: Investigation, Writing – review & editing. JN: Methodology, Writing – review & editing. YQ: Methodology, Writing – review & editing. LG: Data curation, Funding acquisition, Supervision, Writing – review & editing. JZ: Data curation, Funding acquisition, Supervision, Writing – review & editing.

## Footnotes

1
https://itol.embl.de/
