# Corrigendum: Isolation of *Limosilactobacillus mucosae* G01 with inhibitory effects on porcine epidemic diarrhea virus *in vitro* from Bama pig gastroenteritis

**DOI:** 10.3389/fmicb.2024.1488274

**Published:** 2024-10-16

**Authors:** Bin Zhang, Haiyan Shen, Hongchao Gou, Nile Wuri, Chunhong Zhang, Zhicheng Liu, Haiyan He, Jingjing Nie, Yunzhi Qu, Letu Geri, Jianfeng Zhang

**Affiliations:** ^1^Key Laboratory of Livestock Disease Prevention and Treatment of Guangdong Province, Institute of Animal Health, Guangdong Academy of Agricultural Sciences, Guangzhou, China; ^2^College of Veterinary Medicine, Inner Mongolia Agricultural University, Hohhot, China

**Keywords:** PEDV, antiviral activity, *L. mucosae* G01, IFN, Bama pig

In the published article, there were two errors in Figures 4C, 5C as published. In Figures 4C, 5C, due to too many pictures in the data processing process, some pictures were repeated. The corrected Figures 4C, 5C appears below.

**Figure 4:**
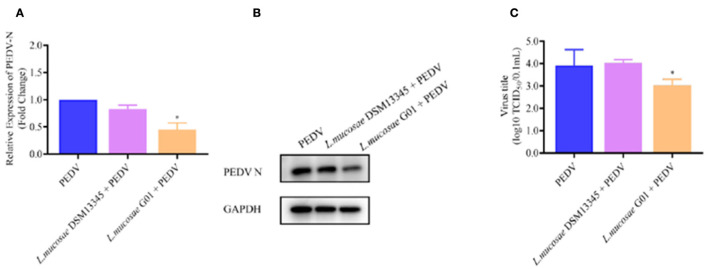
Depicts a comparison of the anti-PEDV effects of 1 MOI *L. mucosae* DSM13345 and *L. mucosae* G01 strains on IPEC-J2 cells. The method was utilized to evaluate the expression levels of the N protein in *L. mucosae* DSM13345 and *L. mucosae* G01 following 2 h interaction with IPEC-J2 cells, subsequent addition of PEDV-HZ, and after 24 h cell supernatants were harvested. Cell lysate containing PMSF was added for nucleic acid extraction and Western Blot assay. **(A)** RT-qPCR method was used to detect *L. mucosae* DSM13345 and *L. mucosae* G01 samples PEDV N mRNA expression level. **(B)** The quantification of N protein expression levels was performed through Western blot analysis. PEDV N is the viral content of the N nucleocapsid, and GAPDH is an internal reference for harvested cells. **(C)** TCID_50_ analysis cell supernatant titer. The purple color is *L. mucosae* DSM13345, orange color is *L. mucosae* G01, and blue color is PEDV control group. All assays were performed in triplicates, with three replicates per experiment, and each bar is the mean ± SEM. The asterisk indicates significant differences compared to the control group (*p* < 0.05).

**Figure 5:**
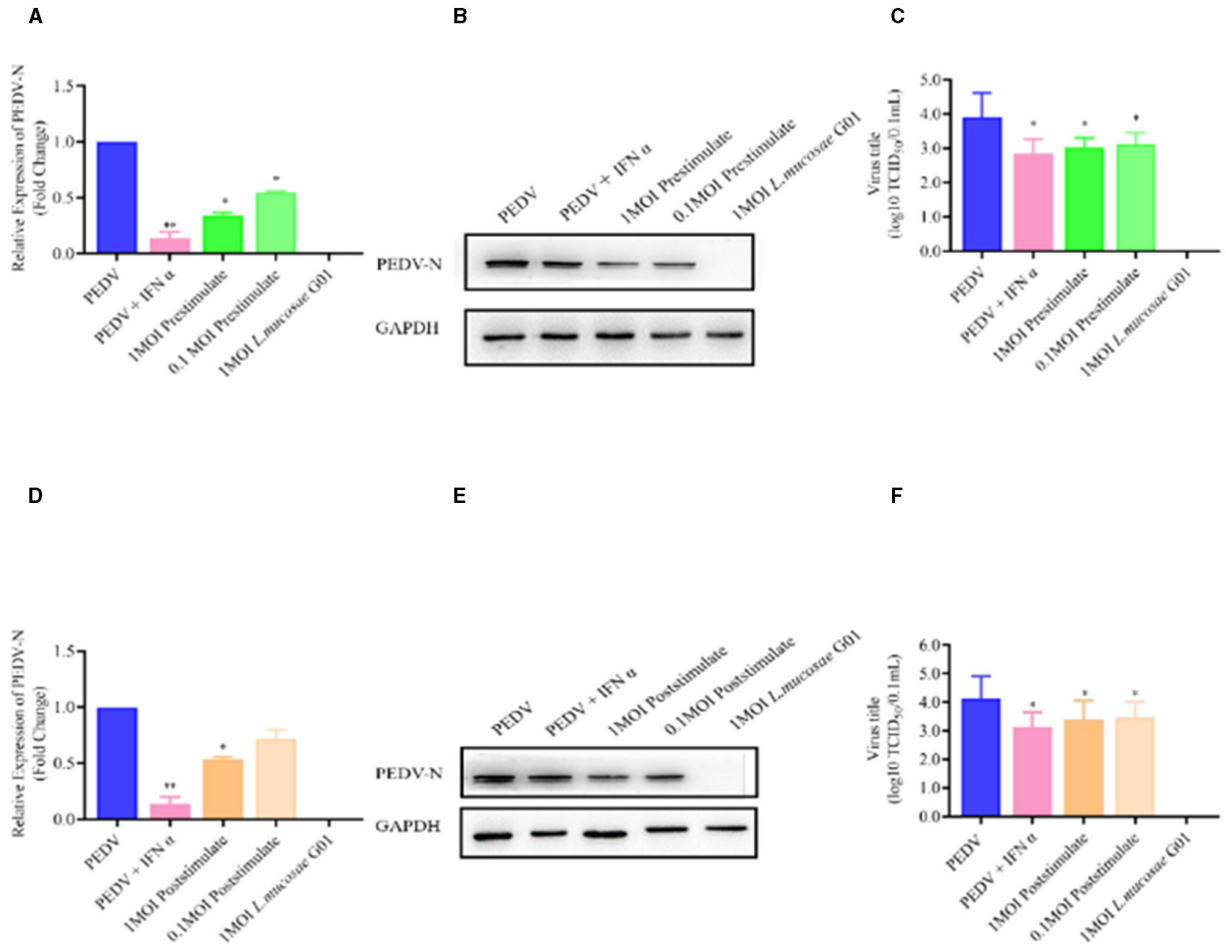
*L. mucosae* G01 inhibits PEDV replication with both prestimulate and post stimulate. Depicts the prestimulation and poststimulation effects of *L. mucosae* G01 strain were demonstrated in IPEC-J2 cells after 24 h with 1 and 0.1 MOI and expression level of PEDV N protein and treat vero cells with a 10-fold dilution of viral supernatant for 72 h. **(A)** Expression content of mRNA levels of PEDV N nucleocapsid protein in response to prestimulation at 1 and 0.1 MOI. **(B)** Expression levels of PEDV N protein in response to prestimulation with at 1 and 0.1 MOI. **(C)** TCID_50_ analysis cell supernatant titer were measured with prestimulation at 1 and 0.1 MOI. **(D)** Expression content of mRNA levels of PEDV N nucleocapsid protein in response to poststimulation at 1 and 0.1 MOI. **(E)** Expression levels of PEDV N protein in response to poststimulation with at 1 and 0.1 MOI. **(F)** TCID_50_ analysis cell supernatant titer were measured with prestimulation at 1 and 0.1 MOI. The experimental groups included PEDV-infected, virus infected after IFN treated cells, cells infected with *L. mucosae* G01 1 MOI and then treated with PEDV, cells infected with *L. mucosae* G01 0.1 MOI and then treated with PEDV, and cells infected with *L. mucosae* G01. Cells infected 0.1 MOI PEDV then treated with *L. mucosae* G01 at 1 and 0.1 MOI. Pink color is PEDV and IFN-α as positive control. Green is the prestimulation group: 1 MOI *L. mucosae* G01 interacted with IPEC-J2 cells followed by 0.1 MOI PEDV, light green is the prestimulation group: 0.1 MOI *L. mucosae* G01 interacted with IPEC-J2 cells followed by 0.1 MOI PEDV, and orange is the treatment group: 0.1 MOI PEDV interacted with IPEC-J2 cells before interacting with 1 MOI *L. mucosae* G01, light yellow is 0.1 MOI PEDV first interacting with IPEC-J2 cells before interacting with 0.1 MOI *L. mucosae* G01 and blue color is PEDV control group. All assays were performed in triplicates, with three replicates per experiment, and each bar is the mean ± SEM. Statistically significant differences between groups are indicated by *p < 0.05 or **p < 0.01.

The authors apologize for this error and state that this does not change the scientific conclusions of the article in any way. The original article has been updated.

